# Immunoproteomic Identification and Characterization of *Leishmania* Membrane Proteins as Non-Invasive Diagnostic Candidates for Clinical Visceral Leishmaniasis

**DOI:** 10.1038/s41598-018-30546-y

**Published:** 2018-08-14

**Authors:** Sarfaraz Ahmad Ejazi, Anirban Bhattacharyya, Somsubhra Thakur Choudhury, Sneha Ghosh, Abdus Sabur, Krishna Pandey, Vidya Nand Ravi Das, Pradeep Das, Mehebubar Rahaman, Rama Prosad Goswami, Nahid Ali

**Affiliations:** 1 Infectious Diseases and Immunology Division, CSIR-Indian Institute of Chemical Biology, Kolkata, India; 2 Department of Clinical Medicine, Rajendra Memorial Research Institute of Medical Sciences, Patna, India; 3 Department of Molecular Biology, Rajendra Memorial Research Institute of Medical Sciences, Patna, India; 4 Department of Tropical Medicine, School of Tropical Medicine, Kolkata, India

## Abstract

Visceral leishmaniasis (VL), a potentially fatal disease is an outcome of infection caused by the parasite *Leishmania donovani*. The clinical diagnostic tests for this disease are still related to invasive tissue aspiration or serological immunochromatography. Advancements in immunoproteomics such as two-dimensional gel electrophoresis, mass spectrometry, B cell epitope prediction, and peptide synthesis have enabled researchers to discover newer biomarkers for disease diagnosis. In this study, we have screened several urine-reactive leishmanial membrane proteins as potential biomarker candidates. In the immunoblot assay, three proteins 51, 55 and 63 kDa showed 100% reactivity to the urine of 47 VL patients and nonreactive to 18 healthy and other diseases. Mass spectrometry revealed the identity of 51, 55 and 63 kDa proteins as elongation factor 1α (EF1-α), α-tubulin, and glycoprotein 63, respectively. B cell reactive epitopes of these proteins were mapped through bioinformatic tools and one epitope from each protein that had the highest score were synthesized. All the three native electroeluted proteins and their corresponding synthetic peptides were tested through ELISA for reactivity with VL and control urine samples. While all three demonstrated good reactivity, the diagnostic performance of EF1-α was the best. Our findings illustrate the use of urine-based proteomic approach for biomarker discovery in non-invasive clinical diagnosis of VL.

## Introduction

Progress in the development of newer antigenic candidates for diagnosis as well as vaccination in visceral leishmaniasis (VL) is largely hampered due to our limited and insufficient knowledge of complex immune responses. Several *Leishmania* proteins have been identified for diagnostic tests. Selection of proteins that have good immunogenicity for B lymphocytes could be an advantage in the diagnosis of VL. Earlier, several vector host and parasite proteins have been identified and evaluated for antibody response within the human host. Heat shock protein (HSP) 83, rGRP78 and HSP70 are some of the most conserved evolutionary proteins identified for serodiagnosis of VL^1^. Leishmanial histone proteins such as H2A, H2B, H3, and H4 have also been identified and tested^2^. Protein A2 from amastigotes has been investigated for antibody response against *L*. *donovani* infection in India, Sudan and America with variable sensitivities^3,4^. Several other nuclear proteins such as rlepp2 and rpaplee22^5,6^, ribosomal proteins rLiP2a, rLiP2b and rP20^7,8^, enzymes rCysteine proteinase and rSignal peptidases^9,10^, and other antigens such as rORFF and Q protein have been studied for diagnostic purposes^11,12^. Antigens with molecular masses of 116 kDa, 72 kDa, 66 kDa and 36 kDa have been used as the biomarker for VL in many earlier studies^13,14^. Most of the proteins identified as diagnostic candidates have been screened for serodiagnosis and limited to laboratory scale validation only.

Genome sequence accessibility of *L*. *donovani* has helped in the study of the expression of genes and proteins by multiple immunoproteomic approaches such as 2D-gel electrophoresis, mass spectrometry and B cell epitope mapping. Immunoproteomics permit the researchers to determine parasite-specific proteins, their interactions with host cells and then specific immune responses during infection. For serological diagnosis of VL, *L*. *chagasi* derived recombinant kinesin-related antigen, rK39 is widely used commercially. However, rK39 antigen often shows cross-reactivity with endemic healthy controls^15^. This antigen has better sensitivity and specificity in the Indian subcontinent as compared to the East African countries and South America^16^. In the last decade, several newer antigens have been identified and characterized for serological diagnosis of VL. The immunodominant domain of kinesin antigen rKE16 has been cloned from an Indian *L*. *donovani* clinical isolate. 100% sensitivity and specificity have been reported with this antigen in Old World VL countries such as India, Pakistan, China, and Turkey^17^. In a further study rKE16 showed comparable sensitivity (96.6%) and specificity (96.2%) with rK39 antigen in India. However, the performance was weaker compared to rK39 in Sudan and France^18^. A fusion protein, rK28 has been generated from three *L*. *donovani* proteins having homology with K39, K26 and K9 of *L*. *infantum*^19^. In this study rK28 RDT showed better sensitivity (94.5%) and specificity (97.6%) with Sudanese serum samples^20^. Another kinesin antigen rKLO8 has been identified and expressed from autochthonous *L*. *donovani* strain in Sudan^21^. The sensitivities, 98%, 96.2% and 100%, and specificities, 100%, 96.06% and 81.85% for rKLO8 have been reported in Sudan, India, and France, respectively^18^. rKRP42 is another kinesin-related protein that has been reported for diagnostic purpose^22^. Development of novel antigen targets for non-invasive diagnosis of VL is still lacking. In some studies, however, antigens which had been developed for serodiagnosis have also been illustrated for urine reactivity. In one such study in Bangladesh rK28 antigen showed 95.4% sensitivity and 98.3% specificity through ELISA with urine samples^23^. In recent years, alternatively, with the help of bioinformatic tools analysis of even unknown putative protein sequences, their role in infection and B cell epitopes have been predicted and subsequently synthesized for diagnostic tests^24^.

Earlier, we have reported the diagnostic ability of leishmanial membrane antigens (LAg) isolated from promastigote form of *L*. *donovani* strain AG83 (ATCC^®^ PRA-413^™^). Reactivity of this crude membrane antigen with urine antibodies paved the way for non-invasive diagnosis of VL^25^. In this study, by means of immunoproteomic approach seeking more defined antigens we identified several urine reactive components of LAg through electrophoresis, immunoblot and mass spectrometry. The study further sought B cell epitope mapping of selected antigens and their corresponding peptides were synthesized and evaluated for VL diagnosis.

## Results

### SDS PAGE of *Leishmania* membrane antigens LAg

**Figure 1:**
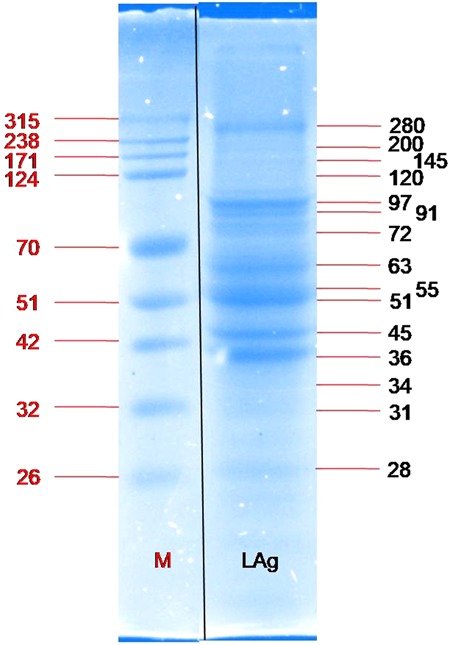
Proteins of different molecular masses separated from *Leishmania* membrane antigens (LAg) on 10% SDS-PAGE. Lane M, molecular weight markers (26–315 kDa); Lane LAg, separated LAg proteins. Both the lanes were part of a single gel.

Earlier we have reported the diagnostic potential of *L*. *donovani* promastigote membrane antigens (LAg) in ELISA (97.94% sensitive and 100% specific) and dipstick (100% sensitive and 100% specific) systems with urine samples^25^. Despite a crude mixture of antigens the sensitivity and specificity of LAg were found to be excellent. Here, we have separated the different protein constituents present in LAg through SDS-PAGE and visualized by Coomassie blue staining. LAg comprises of approximately 15–20 membrane residing proteins ranging in molecular masses from 25–280 kDa. Some of the LAg proteins have good band intensity while others possess comparatively lesser intensity. The major LAg bands visualized with Coomassie were 28, 31, 34, 36, 45, 51, 55, 63, 72, 91, 97, 120, 145, 200 and 280 kDa proteins (Fig. 1).

### Immunoblot assay of urine with LAg proteins

**Figure 2:**
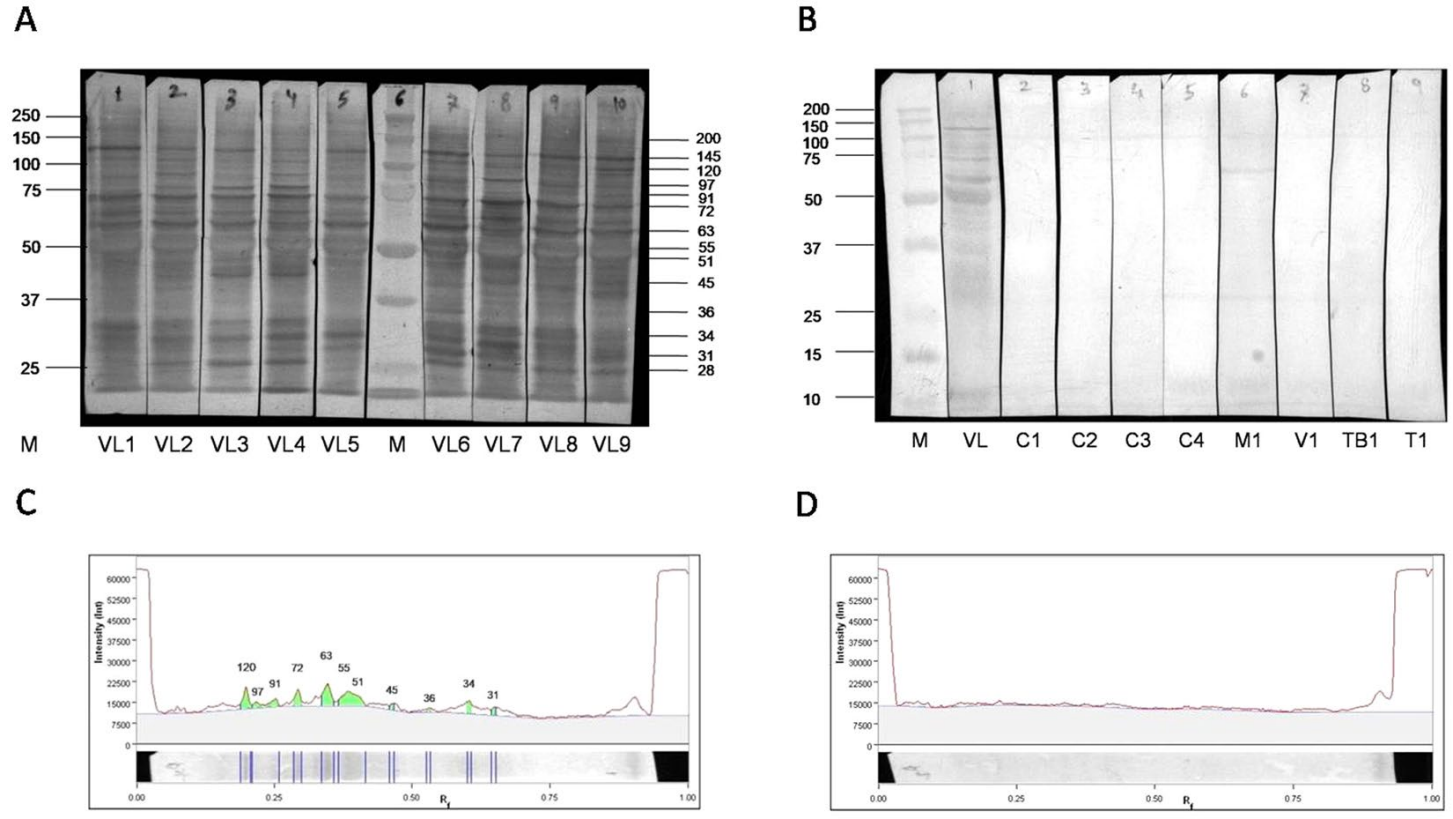
Immunoblot assay of *Leishmania* membrane proteins, LAg with **(A)** 9 infected urine samples (VL1-VL9) and **(B)** 8 non-VL urine samples including non-endemic healthy controls (C1&C2), endemic healthy controls (C3&C4), malaria (M1), viral fever (V1), tuberculosis (TB1) and typhoid (T1) with an infected VL urine sample (VL). Molecular weight marker lane, M, is marked in the left in kilodaltons. Corresponding blots in part A have been cut, treated and arranged from a single gel. Similarly blots in part B belong to a single gel. Images of reactive *Leishmania* membrane proteins to infected **(C)** and healthy **(D)** urine samples were obtained through Image Lab software and urine reactive proteins were marked as peaks in kDa.

We further evaluated the reactivity of different LAg proteins with urine samples through immunoblotting. A total of 47 samples of *L*. *donovani* infected patients were used to study the anti-leishmanial antibodies in their urine against different LAg proteins. Out of the fourteen proteins of LAg observed in immunoblot, six proteins corresponding to relative molecular masses 34, 51, 55, 63, 72 and 120 kDa showed 100% reactivity with all active VL urine (Fig. 2A). Other frequently recognized proteins were of molecular masses 28, 31, 36, 45, 91, 97, 145 and 200 kDa. The data indicated that some of these proteins are more immunogenic than others and can be selected for utilization as future selective diagnostic candidates. The percent recognition of each protein tested with 47 urine VL cases is tabulated in Table 1. Urine reactivity of LAg was also tested with non-leishmanial urine samples for cross-reactivity. Eighteen urine samples from five endemic and non-endemic healthy controls and two each for malaria, viral fever, tuberculosis, and typhoid were tested in the immune blot assay. Antibodies specific to the proteins 28, 31, 34, 36, 45, 51, 55, 63, 72, 91, 97, 120, 145 and 200 kDa, which were present in infected urine were completely absent in healthy controls and other diseases (Fig. 2B). However, cross-reactive bands at 26 and 102 kDa were present in some controls.

**Table 1:** Percent recognition of LAg proteins against VL urine samples.

Molecular mass of LAg proteins (kDa)	200	145	120	97	91	72	63	55	51	45	36	34	31	28
Recognition with *Leishmania* infected urine samples (%)	31.9	48.9	100	96.5	98.2	100	100	100	100	72.3	95.7	100	59	63.8

### Immunoreactive protein band analysis

To estimate the accurate position of the immunoreactive LAg proteins against *Leishmania*-infected urine, blots were analyzed with the Image Lab software in gel doc Instrument. The software showed the intensity of each band in the blot with relative molecular mass, relative front (R_f_) value, band percentage and the lane percentage occupied by each band. With a typical VL urine sample, eleven proteins of molecular masses 31, 34, 36, 45, 51, 55, 63, 72, 91, 97 and 120 kDa were identified (Fig. 2C) with the software. Bands of 51, 55 and 63 kDa showed the highest intensity by volume with approximately 20, 28 and 20% contribution in the whole antigen, respectively (Supplementary Table S1). Cross-reactivity of LAg with healthy urine sample was also analyzed. None of the components of LAg were detected by the software in healthy urine sample (Fig. 2D).

### Urine reactivity of LAg after cure

**Figure 3:**
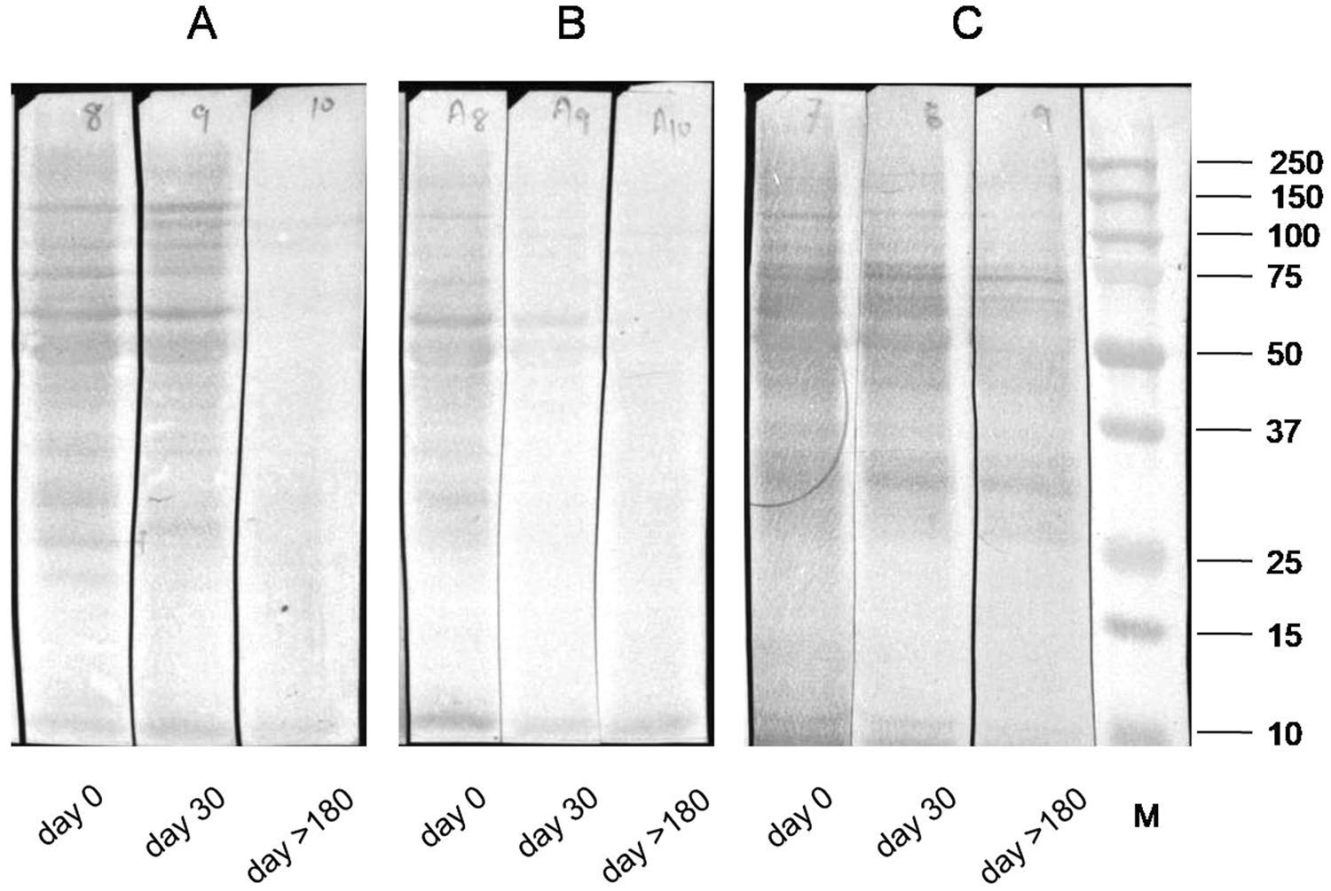
*Leishmania* membrane proteins immunoblotted with paired urine samples before treatment started (day 0), after one month (day 30) and after six months (day > 180) of treatment. Samples were collected from a longitudinal study of 18 VL patients at three time points. Figure shows a representative pattern of reactivity during treatment. **(A)** Urine antibodies did not fall after one month but completely disappeared after six months. **(B)** Urine antibodies fell after one month and completely disappeared after 6 months. **(C)** Urine antibodies remain positive after six months. M, molecular weight markers in kDa. The blots of figures A, B and C were cropped from three separate gels.

Immunoblot assay with 54 paired urine samples from 18 *Leishmania* infected patients before treatment started (day 0), 18 cured samples after one month treatment (day 30) and six months (day > 180) follow-up were tested to study the antigenic reactivity of each polypeptide of LAg protein with therapy. In the active phase of infection before the treatment started, all the urine samples were reactive with 31, 34, 36, 45, 51, 55, 63, 72, 91, 97 and 120 kDa proteins. After completing treatment ten urine samples showed a decline in protein reactivity in 30 days (Fig. 3B). In these cases, antibodies reactive to band 31, 34, 36, 45, 51, 91, 97 and 120 kDa completely declined after 30 days of treatment but 55, 63 and 72 kDa proteins remain detectable. However, urine samples of eight cured VL cases showed almost equal reactivity and band intensity at day 30 similar to the active *Leishmania* infection before treatment (Fig. 3A,C). Samples after six months of treatment initiation illustrated disappearance of all the reactive bands completely except in two VL cases where bands of 31, 34, 45, and 72 kDa were found to be present (Fig. 3C). Thus following six months of treatment 16 out of 18 samples showed complete fall in antibody titer against polypeptides of LAg.

We thus observed three types of protein reactivity with urine samples obtained from *Leishmania*-infected patients in the course of treatment. In the majority of the patients, the antibody level did not fall just after therapy but took at least six months for the full disappearance. In some VL cases, urine antibody titer significantly fell during the course of treatment and could not be detected post-treatment. In two cases antibodies were present in the urine even after six months. This could be a signal for relapse or future development of PKDL. This needs more studies for validation.

**Table 2:** Identified proteins of LAg in MALDI ms/ms analysis.

Protein Band	Protein Name	Accession No.	Protein Score
63	Glycoprotein 63	gi|21954464	143
55	α-tubulin	gi|71840353	206
51	elongation factor 1α	gi|146083153	70

### Mass spectrometry of urine reactive *Leishmania* proteins

**Figure 4:**
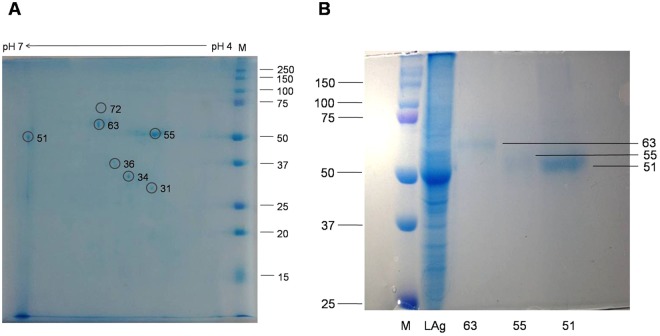
**(A) **Two-dimensional gel electrophoresis of *Leishmania* membrane proteins (LAg). 51, 55 and 63 kDa spots were picked and subjected to tryptic digestion. **(B)** 63, 55 and 51 kDa proteins of LAg were electroeluted and subjected to SDS-PAGE. Corresponding lanes in part B was part of a single gel.

After analysis of each component of LAg for its diagnostic potential and ability to monitor treatment response, we selected three immunodominant antigenic proteins of LAg for identification through mass spectrometry. Urine antibodies against 51, 55 and 63 kDa proteins were found prominently in all VL cases with 100% recognition by urine samples from *Leishmania*-infected patients. Since antibodies against these proteins disappeared completely after six months of treatment, they best suited as candidate biomarkers. In order to identify these three proteins, bands were excised from Coomassie stained SDS-PAGE 2D-gel and subjected to in-gel tryptic digestion (Fig. 4A). The digested proteins were analyzed by MALDI-TOF mass spectrometry and the MS and MS/MS spectra were obtained through MASCOT search against *Leishmania* sequences in MSDB and NCBI databases. Three *Leishmania* proteins which were identified unambiguously from 51, 55 and 63 kDa bands were elongation factor 1α of *L*. *infantum*, α-tubulin of *L*. *donovani*, and glycoprotein or leishmanolysin of *L*. *donovani*, respectively (Supplementary Figs S1–S3). Protein bands identified with their accession no. in database and protein score obtained in MALDI are listed in Table 2.

### RNA sequence expression profiling of selected LAg proteins

In *Leishmania* infection parasite enters into the human bloodstream by the bites of sandflies, the carriers of the promastigote form. These then transform into the amastigote in the peripheral macrophages^16^. Several studies have shown the increased level of antibodies in human sera against both promastigote and amastigote antigens^26^. Presence of promastigote antigen-specific antibodies in the active phase of *L*. *donovani* infection and even after treatment is possible if these antigens are common in both the forms of the parasite^27^. We have shown the reactivity of several *L*. *donovani* promastigote antigens (LAg) with VL urine antibodies. We next investigated the presence of LAg proteins, elongation factor 1α (EF1-α), α-tubulin, and glycoprotein 63 (GP63) in the amastigote stage of the parasite in both virulent and avirulent forms. Intracellular amastigotes from infected peritoneal macrophages of mice after 12 h post-infection were used for RNAseq expression profiling of antigens. All three urine reactive LAg proteins, EF1-α, α-tubulin and GP63 which were identified in promastigotes were also found in the amastigote form of *L*. *donovani* through RNAseq profiling. Moreover, the GP63 protein was up-regulated and EF1-α and α-tubulin were down-regulated in virulent amastigotes as compared to the avirulent ones (Table 3).

**Table 3:** RNA Sequence profiling of three LAg proteins in *L*. *donovani* amastigotes.

Proteins name	GP63, leishmanolysin	Alpha tubulin	Elongation factor 1alpha
Gene names	LDBPK_100510	LDBPK_130330	LDBPK_170170
Base mean avirulent	206.55	17918.23	21886.93
Base mean virulent	296.65	14252.97	20677.37
Fold change	1.43	0.79	0.94
Regulation	up	down	down
Functions	Metallo endopeptidase activity	GTPase activity; structural constituent of cytoskeleton	GTP binding; translation elongation factor activity

### Electroelution and urine reactivity of proteins through ELISA

**Figure 5:**
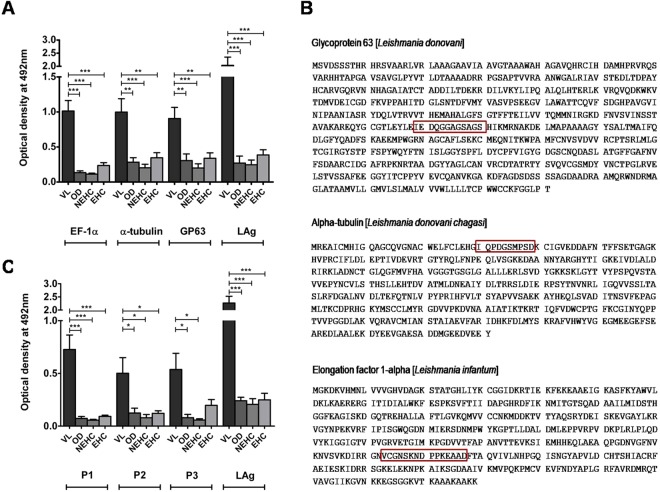
**(A)** Reactivity of three electroeluted antigens GP63, α-tubulin and EF1-α along with crude antigens, LAg, were tested against urine samples of kala-azar infected individuals (VL, n = 20), non-endemic healthy controls (NEHC, n = 10), endemic healthy controls (EHC, n = 10), and other diseases (OD, n = 10) including two of each malaria, viral fever, tuberculosis, typhoid, and pneumonia. **(B)** The whole protein sequence of glycoprotein 63, α-tubulin and elongation factor 1α with their mapped epitopes (red block). **(C)** In comparison to crude membrane antigens, LAg, reactivity of three synthetic peptides P1, P2 and P3 were tested against urine samples of kala-azar infected individuals (VL, n = 40), non-endemic healthy controls (NEHC, n = 20), endemic healthy controls (EHC, n = 10) and other diseases (OD, n = 18) including 6 malaria, 4 viral fever, 4 tuberculosis, 2 typhoid and 2 pneumonia urine samples.

The candidate diagnostic proteins verified by mass spectrometry and RNA sequence profiling, were further evaluated using quantitative ELISA assay. EF1-α, α-tubulin and GP63 proteins were electroeluted from SDS-PAGE gel (Fig. 4B) and subjected to antibody capture ELISA in their native state. Urine antibody response against antigen EF1-α was comparable with LAg reactivity in differentiating active VL cases from other diseases, endemic and non-endemic healthy controls with p values < 0.0001, <0.0001 and 0.0001, respectively (Fig. 5A). However, cross-reactivity with non-VL healthy and other disease controls was found with α-tubulin and GP63. Therefore, out of the three electroeluted native antigens, EF1-α appears to have better diagnostic ability using urine samples.

### B cell epitope mapping of selected *Leishmania* antigens

Since most of the antigens are conserved throughout evolution, crude and native antigens often have a chance of cross-reaction with other symptomatically similar infections. To select specific sequences that are different from sequences of other closely related infective organisms as well as the parasite hosts B cell epitope sequences were derived from the whole antigen. B cell epitopes may recognize definite antibodies thus allowing diagnosis with more specificity. In this study, selected proteins of *L*. *donovani*, EF1-α, α-tubulin, and GP63 were scanned separately for their potential B cell epitope identification. Epitopes were predicted from the whole protein sequence of antigens in NCBI database search through accession no. that were obtained by mass spectrometry MS/MS. The sequence of B cell epitopes for each antigen was acquired in BepiPred program which had the highest score. Epitopes *VCGNSKNDPPKEAAD* from EF1-α, *IQPDGSMPSD* from α-tubulin, and *IEDQGGAGSAGS* from GP63 protein were obtained and selected from the software. Subsequently, each B cell peptide was allowed for the determination of possible hydropathy of epitopes through Expasy software (Supplementary Fig. S4). All epitopes were shown as a surface region of the globular protein. Fig. 5B shows the B cell epitopes of EF1-α, α-tubulin, and GP63 in their whole protein sequences.

### Immunoreactivity of synthetic peptides of B cell epitopes

To select potentially specific target antigens for non-invasive diagnosis of VL we synthesized three B cell epitopes obtained from the above study. B cell epitope of antigens EF1-α, α-tubulin and GP63 were named Peptide1, Peptide 2 and Peptide 3, respectively. Synthesized B cell linear peptides are unfolded structures and might be useful for possible antibody binding in immunodiagnosis. These peptides were tested against urine samples from VL patients, healthy controls, and other disease controls. Amongst all synthetic peptides, peptide P1 that was mapped from protein EF1-α showed significantly higher reactivity with *Leishmania* infected urine as compared to the non-VL control urine samples, similar to LAg. The p values for antibody titer in VL urine against Peptide P1 were found to be 0.0009, 0.0005 and 0.0007 in comparison to endemic healthy controls, non-endemic healthy controls and other disease urine samples, respectively. Peptides P2 and P3, however, showed lower significant reactivity (p values 0.03, 0.02, 0.04 & 0.07, 0.01, 0.02) with VL urine as compared to endemic healthy controls, non-endemic healthy controls and other disease controls, respectively (Fig. 5C).

## Discussion

Antibody detection methods have provided great ease and simplicity in VL diagnostics in comparison to the gold standard tissue aspiration test. Use of leishmanial antigens for diagnostic purposes has been found effective with almost comparable and even better sensitivity and specificity than tissue aspiration. Most of the antigens discovered for VL diagnosis are based on serological assays. However, these antigens are often not well characterized and are either crude or semi-purified such that they often show false positive results or cross-reactivity with other similar infections. Parameters such as sensitivity, specificity, stability, and reproducibility might influence the performance of the antigen to be a good diagnostic candidate. In VL, the commonly used antigen for rapid diagnosis is rK39, a single cloned antigen isolated from *L*. *chagasi* parasite and expressed in a bacterial expression system. However, the performance of this recombinant antigen is variable in different endemic regions and often shows cross-reactivity with endemic healthy controls^15,16^.

Availability of newer methodologies such as protein profiling through 2D gel electrophoresis, protein identification and sequence analysis by mass spectrometry and other immunoproteomic approaches of reactive epitope prediction for synthetic peptides have boosted up the strategies for development of novel diagnostic antigens. Therefore, a well defined new generation antigen based on recombinant proteins or peptides is the need for non-invasive diagnosis of VL. Unlike sera, very few studies have been performed to determine the reactivity of leishmanial antigens against urine antibodies. Nevertheless, most of the antigens that were studied with urine samples have been initially developed for serum-based assays. To the best of our information, this is the first study where the immunoproteomic approach was used to identify and evaluate the defined antigens specific to urine antibodies of VL.

In this study, we have identified fourteen proteins of *L*. *donovani* membrane antigens which demonstrate immunoreactivity with urine antibodies of VL patients. In immunoblot assay and subsequent analysis with Image Lab software six peptides of molecular masses 34, 51, 55, 63, 72, and 120 kDa showed 100% reactivity with VL urine samples. After one month of treatment 31, 34, 36, 45, 51, 91, 97 and 120 kDa proteins showed recognition only in 44% of the samples. However, after six months of cure recognition goes down to 12% for 31, 34, 45 and 72 kDa proteins whereas reactivity with 36, 51, 55, 63 and 120 kDa proteins was not observed. Therefore, proteins with molecular masses of 51, 55 and 63 were selected for further studies. These proteins can be useful not only in the specific diagnosis of VL using urine samples but also for monitoring treatment response.

Through mass spectrometry 51, 55 and 63 kDa bands were identified as elongation factor 1α of *L*. *infantum*, α-tubulin of *L*. *donovani*, and glycoprotein of *L*. *donovani*, respectively. Glycoprotein 63 (GP63) is the major zinc metalloprotease of *L*. *donovani* and is conserved in all *Leishmania* species^28^. GP63 promotes infection and survival in the host cells. It has been shown that *Leishmania* alters host cell signaling through GP63 interaction which is involved in the uptake of the parasites by the host macrophages^29,30^. Persistence of GP63 specific antibodies in the serum of VL patients after the cure has been reported which suggests its role in life-long immunity in cured individuals^31,32^. We have shown the presence of GP63 specific antibodies in the urine samples of all kala-azar patients. Anti-GP63 antibodies were found in most VL cases after treatment which completely disappeared after six months. Protein α-tubulin is one of the constituents of microtubule networks present in *Leishmania*. It is associated with cell shape, locomotion, and division of the parasite. In several studies, α-tubulin has been shown to be immunogenic in VL patients’ sera and implicated as a vaccine candidate for kala-azar^33^. A high intensity of anti-β tubulin antibodies was observed in urine of VL infected patients through immunoblot which disappears after six months of treatment. EF1-α plays a major role in protein synthesis and their assembly. Belonging to GTP-binding protein, it is highly conserved, and engages in protein translation. EF1-α has also been reported as a virulence factor in *Leishmania* where it diffuses into the infected host macrophages and deactivates several pathways^34,35^. EF1-α has been found to be involved in the humoral immune response by inducing antibodies against *Leishmania*^36^. We have observed the presence of EF1-α specific antibodies in all the urine samples from VL patients. These antibodies persist in most VL cases just after treatment but fall significantly and become negative after six months.

We have always been eager to know why promastigote specific antibodies reside in the human host for so long even after treatment while within 24 h of infection promastigotes transform into amastigotes. This is possible only when amastigotes also share these antigens, thus promoting antibody production throughout infection. We have confirmed this by transcriptome analysis of virulent and avirulent amastigote proteins. We found all the three proteins of promastigote, EF1-α, α-tubulin, and GP63, are present in the amastigote stage of parasite too. GP63 protein was found up-regulated in the virulent form of amastigotes. This suggests a direct role of these proteins in parasite infection and progression. EF1-α and α-tubulin proteins were found to be down-regulated in the virulent stage of amastigotes, suggesting its role in parasite survival and normal growth maintenance in adverse conditions. Therefore, the function of these proteins in *Leishmania* infection supports them as diagnostic biomarkers.

To further investigate the quantitative reactivity of EF1-α, α-tubulin, and GP63 proteins were electroeluted and subjected to antibody capture ELISA. All the three chosen proteins helped distinguish active VL from other diseases and healthy controls. However, EF1-α showed better specificity than α-tubulin and GP63 in their native state. Native proteins either purified or recombinant ones may carry amino acid sequences that can cross-react with other infections. B cell epitope prediction for pathogenic organisms has widened the variety of antigenic peptides and has helped to improve the antigenic immunogenicity. In the current study we have predicted and synthesized three epitopic peptides P1, P2 and P3 from *L*. *donovani* antigens EF1-α, α-tubulin and GP63, respectively. Synthetic peptide sequences were evaluated for antigenicity and diagnosis of VL with urine samples. Peptide P1 demonstrates best sensitivity and specificity among the three synthetic peptides and comparable to LAg. Results with synthetic peptides might be improved further with combinations of two and more peptides and can be used in a point of care test in future studies (already started). Synthetic peptides in VL diagnosis could circumvent the dependency of native antigens which often differ in protein content during batch to batch production and also in reactivity in different endemic regions. However, synthetic peptides are rather simple and do not show much variation in the assay. In conclusion, in this study, we identified and evaluated urine antibody specific defined peptides that can be used for the development of a non-invasive diagnosis of VL.

## Methods

### Sample collection

Urine samples for this study were obtained from School of Tropical Medicine (STM), Kolkata and Rajendra Memorial Research Institute of Medical Sciences (RMRIMS), Patna. Random urine samples were collected from parasitologically confirmed kala-azar patients before the actual treatment started (47 patients at day 0), after completion of treatment (18 patients at day 30) and after at least six months of treatment commencement (18 patients at day more than 180). Urine samples were also collected from 20 non-endemic and 10 endemic healthy controls each along with 20 other diseases samples including 6 malaria, 4 viral fever, 4 tuberculosis, 4 typhoid and 2 pneumonia. After collection, 0.1% sodium azide (NaN_3_) was added to each sample as a preservative and stored at 4 °C until the study was done.

### Ethical approval

Ethical approval for the human study was acquired from the Ethical Committee of CSIR-Indian Institute of Chemical Biology, Kolkata, India along with STM, Kolkata and RMRIMS, Patna. The experiments conducted for the study were carried out with the regulations and guidelines of the committee. Written informed consent in prescribed format was received from all the participants before sample collection. The aim of the study and privacy of participants were mentioned in the consent form. Ethical approval was also taken from the Institute’s animal ethical committee (IICB, Kolkata).

### SDS-PAGE

Leishmanial antigen, LAg was isolated from strain AG83 (ATCC^®^ PRA-413™) of *L*. *donovani* promastigotes as described elsewhere^37,38^. Antigen LAg (10 μg/lane) was resolved in 10% sodium dodecyl sulfate-polyacrylamide gel electrophoresis (SDS-PAGE) in a Mini-Protein II apparatus (BioRad Laboratories, USA). Briefly, prior to loading protein was denatured in sample buffer (2% SDS, 10% glycerol, and 0.025% bromophenol blue in 60 mM Tris-HCl, pH 6.8. 5% 2-mercaptoethanol) in a boiling water bath for 5 min. 10% acrylamide for separating and 4% acrylamide for stacking were used with 1 mm gel thickenings. Finally, proteins were run at constant voltage (60 V). Markers (BioRad Laboratories, USA) of known molecular weights were used for comparison. Protein bands were visualized by Coomassie brilliant blue. Molecular mass of proteins presented in LAg were determined by the R_f_ values obtained with standard marker proteins. Additionally, protein gel was also analyzed with Image Lab software to automatically define the molecular weights in reference to the standard proteins.

### 2D-gel electrophoresis

LAg proteins were also separated in two dimensional (2D) gel electrophoresis. The first dimension of isoelectric focusing (IEF) was carried out with 18 cm strip, pH 4–7 (BioRad Laboratories, USA). The strip was rehydrated (4% CHAPS, 2% ampholyte, 8 M urea, 30 mM DTT and bromophenol blue) overnight at room temperature followed by IEF with Mutiphor II and a DryStrip kit (GE Healthcare, Germany). The strip was then equilibrated for 15 min in buffer containing 6 M urea, 2% SDS, 20% glycerol, 1% DTT, and 5 mM Tris-HCl, pH 8.8. This time the strip was equilibrated with 2.5% iodoacetamide. For the second dimension, 1.5 mm thick 12% SDS-PAGE was run and protein spots were visualized with Coomassie brilliant blue.

### Immunoblot assay

The PAGE-separated proteins were electrophoretically transferred to nitrocellulose membranes at 85 V/cm for 75 minutes in a transblot apparatus (BioRad Laboratories, USA). Transferred LAg proteins on nitrocellulose membrane were confirmed and visualized with Ponceau dye and different lanes were cut into strips and marked for reaction with different urine samples. Each nitrocellulose membrane strip was then blocked overnight in 5% BSA at 4 °C in 100 mM TBS containing 0.1% Tween 20. The next day strips were washed once with washing buffer (100 mM TBS & 0.05% Tween-20) and then incubated with diluted urine samples (1:5 in washing buffer) for 2 hr at room temperature with constant stirring. After incubation with urine, strips were then washed two times for five minutes in wash buffer and dipped in diluted (1:2000) peroxidase-conjugated goat anti-human IgG for 90 min at room temperature. Strips were then washed two more times with the same wash buffer and the third wash was with 100 mM TBS only (without Tween-20). After washing the strips were spread with Luminol and H_2_O_2_ and pictures were taken in ChemiDoc chemiluminescent apparatus (BioRad Laboratories, USA). In another set of an experiment after the last wash strips were incubated for 5 min in freshly prepared substrate solution of 0.05% of DAB + 0.05% of H_2_O_2_ in 100 mM TBS. Finally, the strips were rinsed with distilled water and dried. Image of the membrane blots was captured and analyzed in Image Lab software (version 54.2.1).

### Mass spectrometry

LAg proteins were separated by 2D gel electrophoresis and stained with Coomassie blue dye. Proteins of molecular mass 51, 55 and 63 kDa were excised from the gel through pipette tip as 1 mm^3^ diameter pieces. The in-gel tryptic digestion of proteins was carried out according to the manufacturer’s manual (Pierce, Rockford). Briefly, gel pieces were destained completely in ammonium bicarbonate and acetonitrile solution followed by alkylation and reduction by tris, 2-carboxyethyl phosphine (TCEP) and iodoacetamide, respectively. The gel pieces were digested in 10 ng/µl concentration of enzyme trypsin in ammonium bicarbonate solution and incubated overnight at 37 °C. The peptides (0.5 µl) were co-crystallized with matrix (5 mg/ml CHCA, 5% ACN and 0.1% TFA) in the metal plate well of ABSciex 4800 mass spectrometer (Applied Biosystems). Both mass spectrometry (MS) and MS/MS spectra were obtained by matrix-assisted laser desorption ionization–time of flight (MALDI-TOF/TOF) mass Spectrometer. Database searching for protein identification was performed with mass spectrometry data using GPS Explorer (Applied Biosystems) software with MASCOT (Matrix Science) search engine.

### Electroelution of proteins

The LAg proteins of molecular masses 51, 55 and 63 kDa were excised from 10% SDS-PAGE gel stained with Coomassie blue. Excised protein bands were eluted using electrophoresis apparatus (BioRad, Model-422) under running buffer (1% SDS, 0.192 M glycine and 0.025 M Tris) for 4 h at 10 mA. Subsequently, eluted proteins were dialyzed and resuspended in PBS. Finally, proteins were quantified by Lowry’s method and position of the proteins was reconfirmed in SDS-PAGE.

### RNA sequence profiling

Peritoneal macrophages were isolated from healthy BALB/c mice. 3^rd^ to 5^th^ passage of promastigote culture was used for virulent *Leishmania* infection and 25^th^ passage culture was used for avirulent infection in peritoneal macrophages. After approximately 12 h of infection total RNA was isolated and RNAseq profiling was done to compare the expressed genes of virulent and avirulent parasites. Promastigote specific proteins corresponding to their RNAseq and genes were compared against amastigote and database using BLASTp search.

### B cell epitope mapping

The sequence of three *L*. *donovani* proteins, EF1-α, α-tubulin, and GP63 were obtained from NCBI database. All the sequences were mapped by using BepiPred software program with a threshold of 0.35 (http://www.cbs.dtu.dk/services/BepiPred). B cell epitopes predicted for each protein were analyzed further with Expasy for possible hydropathy of the epitopes (http://web.expasy.org/protscale). Selected epitopes were blast searched in NCBI database for similarity analysis with other organisms. B cell epitopic peptide sequences matched by the two programs were selected and synthesized at the Biotech Desk peptide synthesis facility. One peptide was synthesized for each of the three proteins with 96% purity in HPLC.

### Antibody capture ELISA

Antibody capture ELISA with electroeluted proteins and synthetic peptides was performed using 96-well flat bottom ELISA plates (Nunc Maxisorp, Denmark). Briefly, 1 µg/well of electroeluted antigens, EF1-α, α-tubulin and GP63, as well as the crude antigen, LAg were coated in 0.02 M phosphate buffer, pH 7.4 (100 µl/well) and kept overnight at 37 °C. In another set of ELISA 10 µg/well of synthetic peptides P1, P2 and P3 along with antigen LAg were used. Plates were blocked with 1% of bovine serum albumin (BSA) (Sigma, USA) in 200 µl/well of 0.02 M phosphate buffer saline (PBS, pH 7.4) for 2 h at 37 °C. Subsequently, 100 µl/well of urine samples (1:10 dilution in blocking) followed by HRP-conjugated anti-human IgG (Bangalore GeNei, India) (1:4000 dilution) were applied and incubated at 37°C for 1 h. ELISA plates were washed three times with PBS containing Tween-20 in each step. Finally, 0.05% (w/v) o-phenylenediamine dihydrochloride (OPD) (Sigma, USA) as substrate was added in 50 mM phosphate-citrate buffer (100 µl/well) with 0.05% H_2_O_2_ (Merck, Germany). Subsequently, 2 N sulfuric acid (50 µl/well) was used to stop the reaction and the optical density was determined at 492 nm in an ELISA reader (RS232C, Thermo Scientific, MA, USA).

### Statistics

Statistical analyses in this study were performed with the software Graph Pad Prism version 5.0. Two-tailed, Mann-Whitney *U* test, unpaired t-tests were used for comparison of ELISA values of different groups and considered statistically significant if the *P* values < 0.05.

## Electronic supplementary material

Supplementary Information

## Data Availability

All data generated or analyzed during this study are included in this published article (and its Supplementary Information files).
