# Supplementary material for: Immunoproteomic Identification and Characterization of Leishmania Membrane Proteins as Non-Invasive Diagnostic Candidates for Clinical Visceral Leishmaniasis
Source: Sci Rep. 2018 Aug 14;8:12110. doi: 10.1038/s41598-018-30546-y (PMC6092337; doi:10.1038/s41598-018-30546-y)
Supplement: Supplementary file 1 — Supplementary Information [file 41598_2018_30546_MOESM1_ESM.pdf]

Immunoproteomic Identification and Characterization of *Leishmania* Membrane Proteins as Non-Invasive Diagnostic Candidates for Clinical Visceral Leishmaniasis.

Sarfaraz Ahmad Ejazi[1], Anirban Bhattacharyya[1], Somsubhra Thakur Choudhury[1], Sneha Ghosh[1], Abdus Sabur[1], Krishna Pandey[2], Vidya Nand Ravi Das[2], Pradeep Das[3], Mehebubar Rahaman[4], Rama Prosad Goswami[4], Nahid Ali[1]*

[1]Infectious Diseases and Immunology Division

CSIR-Indian Institute of Chemical Biology, Kolkata, India

[2]Department of Clinical Medicine

[3]Department of Molecular Biology

Rajendra Memorial Research Institute of Medical Sciences, Patna, India

[4]Department of Tropical Medicine

School of Tropical Medicine, Kolkata, India

*Corresponding author

E-mail: nali@iicb.res.in

Telephone number: +91 33 2499 5757;

Fax no: +91 33 2473 5197

Table S1. Molecular masses of urine reactive *Leishmania* peptides obtained from Imagelab.

Band No.	Mol. Wt. (kDa)	Relative Front (R_f)	Volume (Int)	Band %.	Lane %
1	120	0.198	5,005,392	14.0	1.5
2	97	0.253	4,432,848	12.4	1.3
3	91	0.271	4,268,157	12.0	1.2
4	72	0.292	3,883,404	10.8	1.1
5	63	0.345	7,100,688	19.8	2.1
6	55	0.386	9,989.280	27.9	2.9
7	51	0.410	8,012,105	19.7	2.0
8	45	0.467	1,141,140	3.2	0.3
9,	36	0.530	678,468	1.9	0.2
10	34	0.605	2,143,680	6.0	0.6
11	31	0.648	1,492,092	4.2	0.4

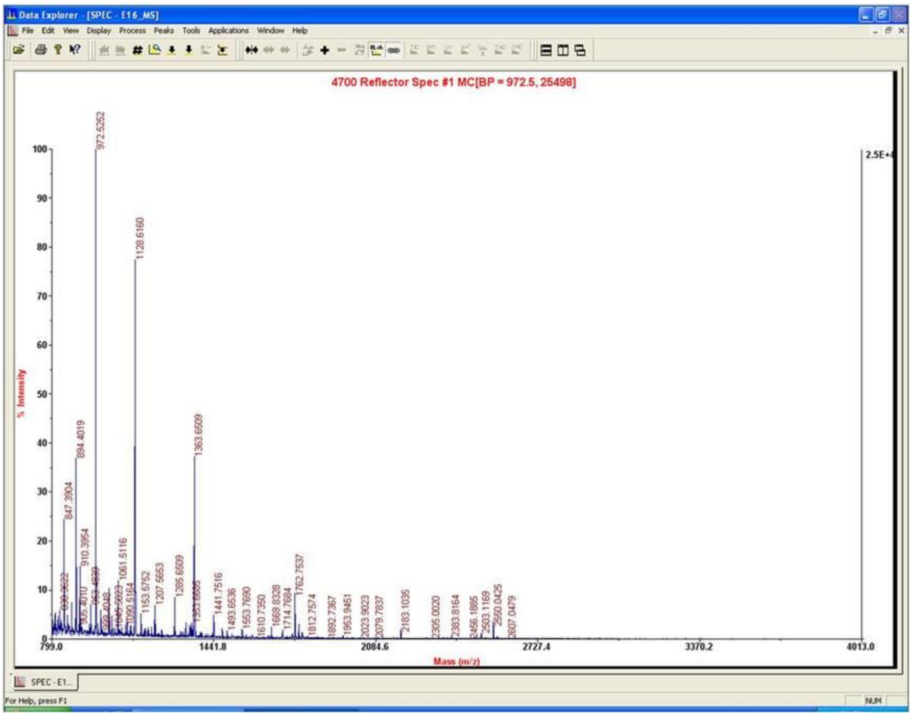

Figure S1. MALDI-TOF spectra of the tryptic fragments obtained from peptide 51 kDa.

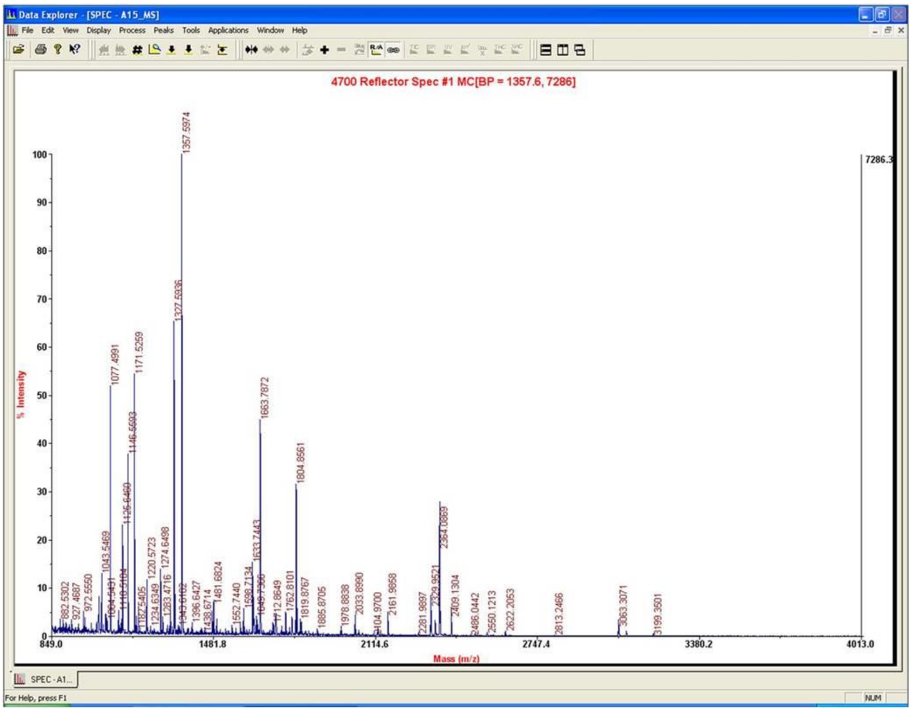

Figure S2. MALDI-TOF spectra of the tryptic fragments obtained from peptide 55 kDa.

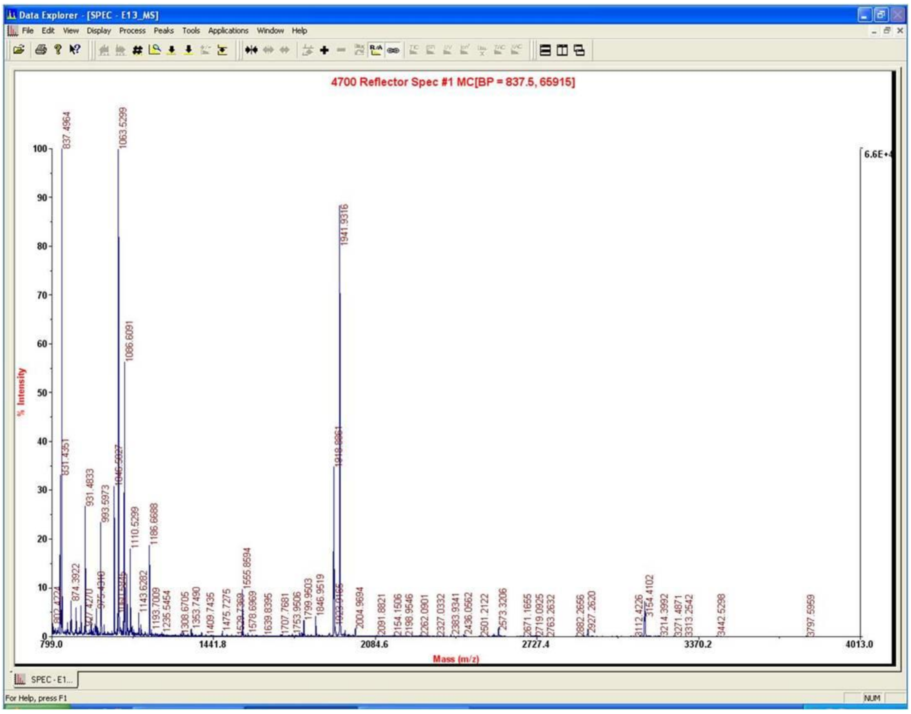

Figure S3. MALDI-TOF spectra of the tryptic fragments obtained from peptide 63 kDa.

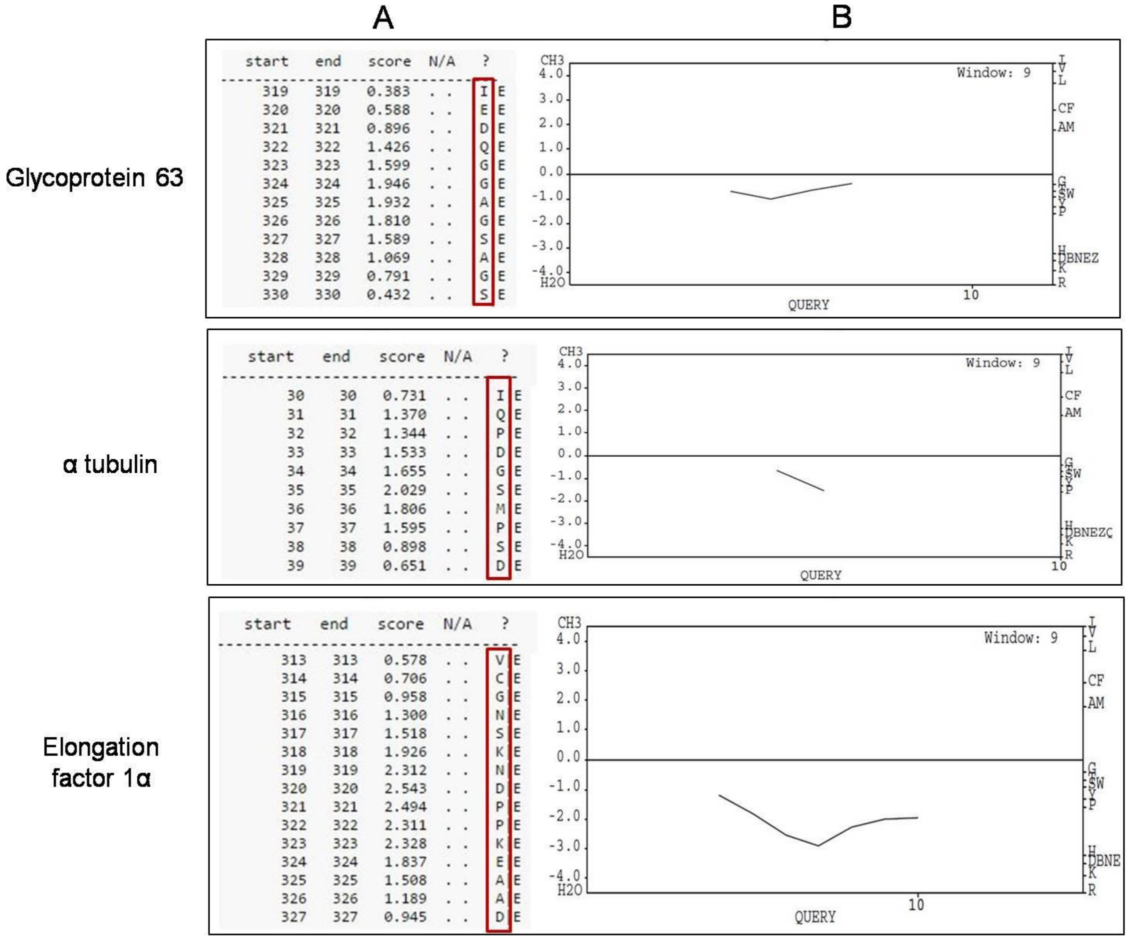

Figure S4. B cell epitope prediction of protein glycoprotein 63, α tubulin and elongation factor 1α in BepiPred software (A) and Kyte & Doolittle hydropathy plot of selected epitopes (B).

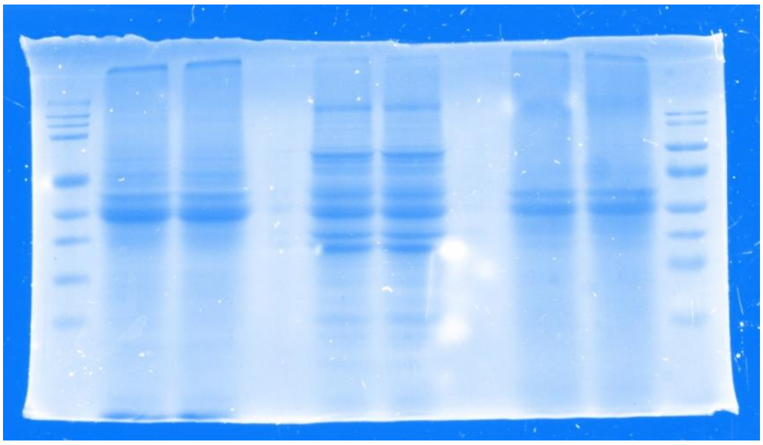

Figure S5. Original gel of figure 1

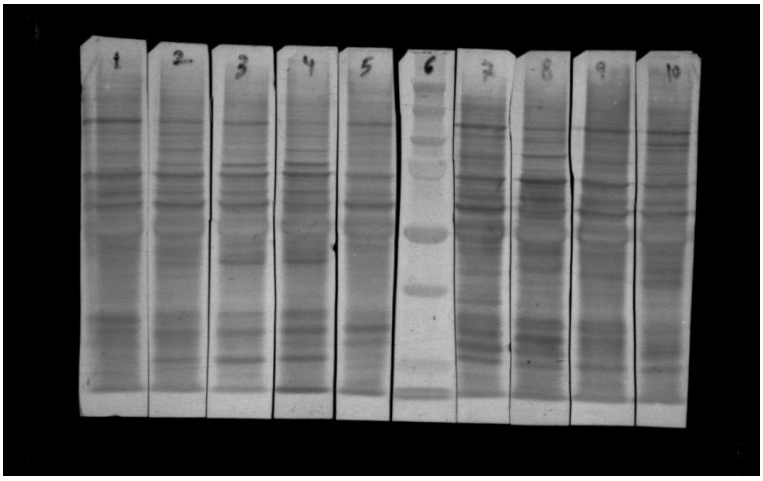

Figure S6. Original blot of figure 2A

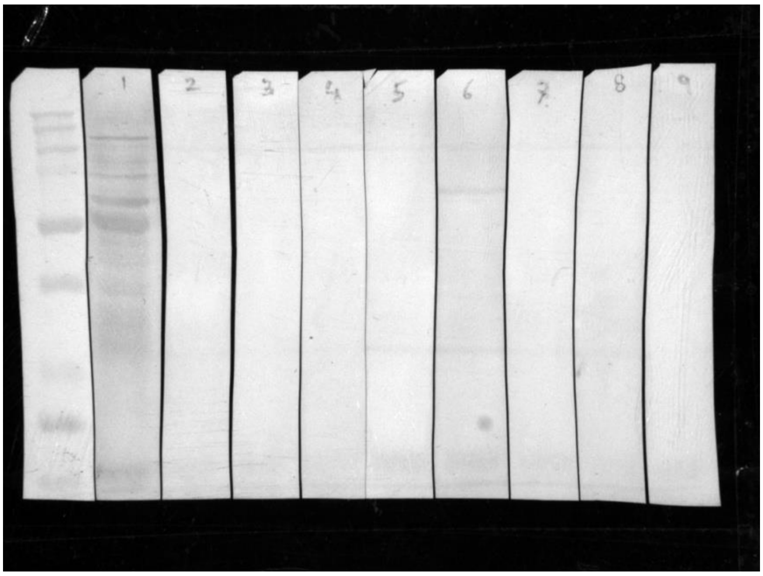

Figure S7. Original blot of figure 2B

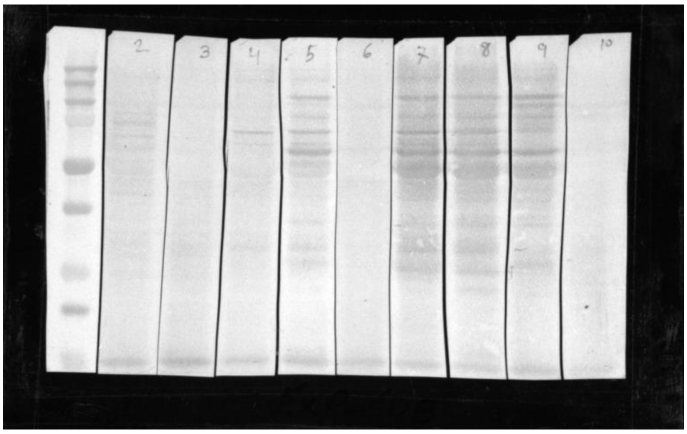

Figure S8. Original blot of figure 3A

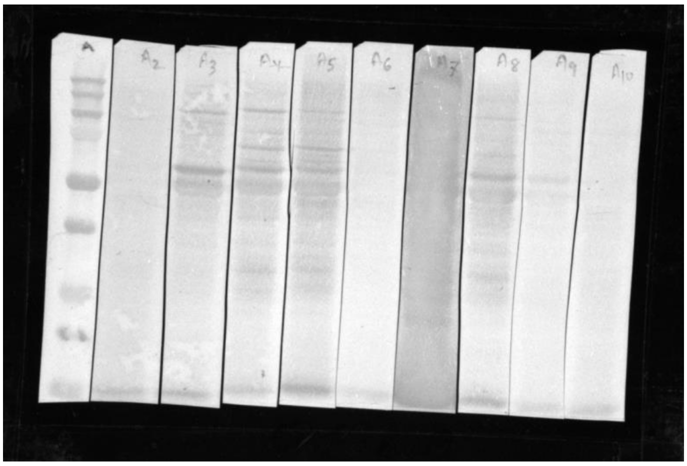

Figure S9. Original blot of figure 3B

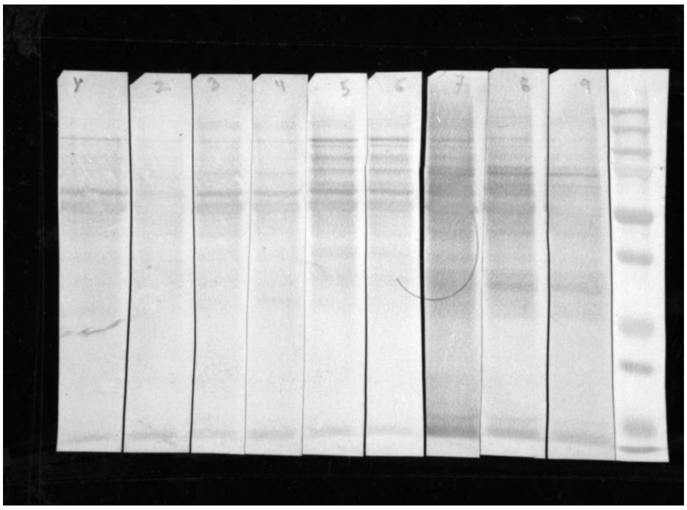

Figure S10. Original blot of figure 3C

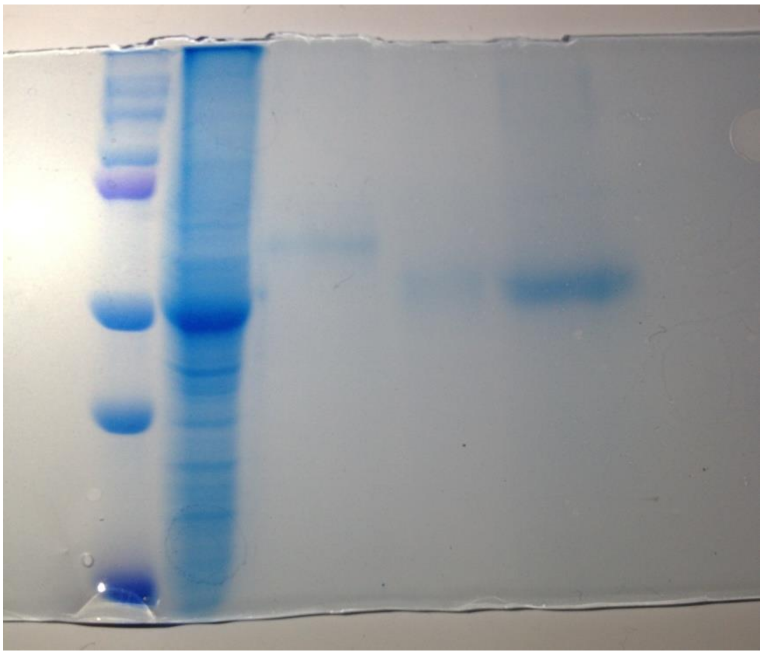

Figure S11. Original gel of figure 4B
